# Resection of pancreatic ductal adenocarcinoma with synchronous distant metastasis: is it worthwhile?

**DOI:** 10.1186/1477-7819-12-347

**Published:** 2014-11-18

**Authors:** Emmanuel Buc, David Orry, Olivier Antomarchi, Johan Gagnière, David Da Ines, Denis Pezet

**Affiliations:** Department of Digestive and HPB Surgery, CHU Estaing - 1, Place Lucie et Raymond Aubrac, 63003 Clermont-Ferrand, France; Department of Oncologic Surgery, Centre Georges François Leclerc, Dijon, France; Department of Radiology, CHU Estaing, Clermont-Ferrand, France

**Keywords:** Pancreatic cancer, Metastasis, Surgery, Chemotherapy, Survival

## Abstract

**Background:**

The purpose of this study is to report prolonged survival in patients with metastatic pancreatic ductal adenocarcinoma (PDAC) managed by chemotherapy and surgery.

**Methods:**

Between January 2009 and August 2013, 284 patients with metastatic PDAC were managed in our oncologic department. Among them, three (1%) with a single metastasis (liver in two cases and interaorticaval in one case) underwent one- or two-stage surgical resection of the metastasis and the main tumor. Perioperative data were recorded retrospectively, including disease-free and overall survival.

**Results:**

The three patients had chemotherapy (FOLFOX or FOLFIRINOX regimen) with objective response or stable disease prior to surgery. Median time between chemotherapy and surgery was 9 (8 to 15) months. Resection consisted in pancreaticoduodenectomy in the three cases. None of the patients had grade III/IV postoperative complications, and median hospital stay was 12 (12 to 22) days. All the patients had postoperative chemotherapy. Only one patient experienced recurrence 11 months after surgery and died after 32.5 months. The two other patients were alive with no recurrence 26.3 and 24.7 months after initial treatment.

**Conclusion:**

Radical resection of PDAC with single distant metastases can offer prolonged survival with low morbidity after accurate selection by neoadjuvant chemotherapy.

## Background

Pancreatic ductal adenocarcinoma (PDAC) is the fourth leading cause of cancer-related deaths in the United States and the sixth in Europe and Japan [1]. Surgery remains the only chance of cure and should be performed when involvement is limited to the pancreatic gland. In cases of advanced disease, such as major vascular involvement, peritoneal carcinomatosis or distant metastasis, palliative treatment is mandatory because surgery is of no benefit while incurring a high risk of complications and patient discomfort [2]. Despite aggressive surgical therapy, only 10 to 15% of patients are eligible for curative resection, which provides a long term survival rarely exceeding 20% at 5 years [3]. The high rate of recurrence after curative resection suggests frequent occult disease or micrometastasis at the time of surgery [4]. For this reason, adjuvant gemcitabine-based chemotherapy has become a standard treatment following resection of PDAC, although the FOLFIRINOX regimen has been recently approved as a new alternative in selected patients [5].

Surgical resection of PDAC with synchronous distant metastases is not mandatory since median survival time is equivalent to that of chemotherapy alone [6]. However, several publications have reported successful resection of PDAC with distant metastases and long-term survival [7–10]. In these observations, neoadjuvant systemic chemotherapy or chemoradiation was administered initially because the disease was considered to be palliative. However, objective response based on imagery and blood markers suggested that occult disease had disappeared, so we decided to perform laparotomy and resection.

Herein, we present a series of three patients with PDAC and distant metastases who underwent pancreaticoduodenectomy (PD) following objective response to neoadjuvant chemotherapy.

## Methods

Between January 2009 and August 2013, 689 patients were investigated in our unit for PDAC. Management of PDAC in our tertiary center is consistent with international recommendations [11].

### Pre-therapeutic assessment

All patients were first assessed using contrast-enhanced computed tomography. In the event of distant metastases or locally advanced disease (involvement of the superior mesenteric artery or the celiac axis), percutaneous or endoscopic ultrasound guided biopsy was performed for histological diagnosis, and chemotherapy was administered based on a multidisciplinary review. Endoscopic or percutaneous stenting was mandatory when total serum bilirubin was >1.5 mg/dL to allow chemotherapy [12]. For resectable patients, preoperative biopsy was not performed routinely, and stenting with a short metallic coverable stent was performed when we estimated that total serum bilirubin would be higher than 15 mg/dL at the time of laparotomy [13].

### Therapeutic strategy

All patients were discussed in our oncological multidisciplinary review board. Details of the therapeutic options were given to the patients, and treatment was started after informed consent. Patients with resectable disease at preoperative evaluation were considered for resection. After abdominal exploration and fresh frozen section, patients with para-aortic lymph node involvement (considered as distant metastases), peritoneal dissemination, liver metastasis or arterial encasement (without prior chemotherapy) underwent a palliative procedure. All other patients were considered eligible for resection. For tumors located in the head of the pancreas, the standard procedure was PD with posterior first approach of the superior mesenteric artery (SMA). For tumors located on the left of the splenoportal confluence, the standard procedure was distal pancreatectomy with splenectomy and lymph node dissection along the left aspect of the celiac axis and the superior mesenteric artery. Extended lymphadenectomy was not performed routinely. Postoperatively, all the patients received adjuvant gemcitabine-based chemotherapy except in case of poor general condition. Patients with locally advanced disease underwent initial chemotherapy based on a multidisciplinary review and, in the event of objective response, surgical exploration and resection when involvement was still limited to the pancreatic gland. Patients with metastatic disease diagnosed either preoperatively or intra-operatively were considered suitable for definitive palliative chemotherapy.

### Oncologic follow-up

All patients were assessed by physical examination, tumor biomarker CA19-9 and computed tomography (CT) scan. Patients with resectable disease were screened every 3 months for the first year and every 6 months thereafter. Recurrences were treated with gemcitabine-based or FOLFIRINOX regimen based on a multidisciplinary review. Patients with advanced disease were assessed after first-line chemotherapy. In cases of objective response, surgical resection was reconsidered. Patients with metastatic disease were assessed after each line of chemotherapy, and the following treatment was decided on after a multidisciplinary review.

## Results

Two hundred eighty-four patients (41.2%) had metastatic disease (199 to the liver, 90 to the peritoneum, 60 to the lung, 42 to the lymph nodes and 36 elsewhere), 261 (37.9%) had locally advanced disease, and 144 (20.9%) were resectable (Table 1). Of the patients with metastatic disease, 3 (1.0%) underwent resection of both the primary tumor and metastases. Clinical and perioperative data are summarized in Table 2.

**Table 1:** **Oncologic data at diagnosis of the 689 patients**

**Entire cohort**	689
**Metastatic** ^**a**^ **(%)**	284 (41.2)
Liver	199 (70.1)
Peritoneum	90 (31.7)
Lung	60 (21.1)
Distant lymph nodes	42 (14.8)
Others	36 (12.7)
**Locally advanced (%)**	261 (37.9)
**Resectable (%)**	144 (20.9)

^a^patients could have several metastatic sites.

**Table 2:** **Clinical data for the three patients**

	Patient 1	Patient 2	Patient 3
Age	65	53.5	60
Sex	M	F	F
BMI	25.7	19.5	21.8
Initial CA19-9	2876	200	34.1
Initial treatment	CT	Surgery	Surgery
Site of metastases	Liver	Interaortocaval nodes	Liver
Number of metastases	Single	Single	Single
Time between first line CT scan and resection	9 months	8 months	15 months
Preoperative CA19-9	34.8	5	17
Type of resection	PD with resection of SMA and PV	PD with resection of PV	PD with resection of SMV
Underlying IPMN	No	No	No
pTNM stage	T4 N0 M1	T3 N0 M1	T3 N1 M1
Retroportal margin	>1 mm	>1 mm	>1 mm
Posterior margin	>1 mm	>1 mm	<1 mm
Anterior margin	>1 mm	>1 mm	>1 mm
Follow-up (months)	32.5	26.3	24.7
Recurrence	Yes	No	No
Disease-free progression (months)	11	/	/

CT, systemic chemotherapy; BMI, body mass index; IPMN, intraductal papillary mucinous neoplasm; PD, pancreaticoduodenectomy; PV, portal vein SMA, superior mesenteric artery; SMV, superior mesenteric vein.

### Patient number 1

**Figure 1:**
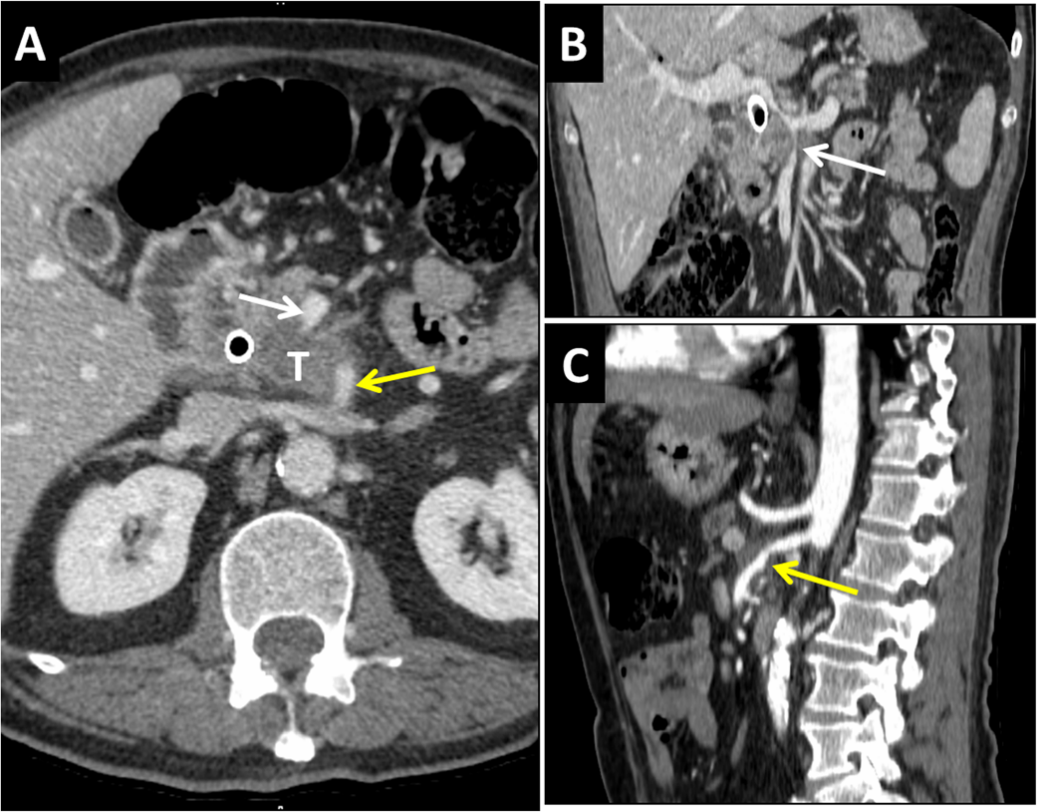
**Computed tomography (CT) scan of patient number 1 showing a tumor of the head of the pancreas (T) with infiltration of both the superior mesenteric vein (SMV) (white arrow) and the superior mesenteric artery (SMA) (yellow arrow). (A)** Axial view; **(B)** frontal view; **(C)** sagittal view.

Patient number 1 was a 65-year-old male with no previous history. Clinical symptoms were abdominal pain and jaundice. Initial CT scan showed locally advanced PDAC (44 mm) with extension to the SMA and SMV (Figure 1). PET scan confirmed the absence of detectable liver metastases. Twelve cycles of neoadjuvant chemotherapy (FOLFOX regimen) were administered. Tumor biomarker CA19-9 returned to normal range at the end of the treatment (2876 to 34.8 UI/L). CT scan evaluation showed stable disease with no distant metastases. Surgery was decided on because of the initial good response to chemotherapy and stable disease after a period of 9 months. At laparotomy, wedge resection of a suspect lesion in segment I was resected with intraoperative frozen section that exhibited no neoplastic cells. PD with superior mesenteric artery (SMA) and portal vein (PV) en bloc resection was then performed. The SMA was reconstructed by termino-terminal end-to-end anastomosis. The PV was reconstructed by end-to-end anastomosis between the superior mesenteric vein (SMV) and the PV, and the splenic vein was re-implanted in the inferior mesenteric vein. The conclusion of the final pathologist’s report was PDAC with portal vein invasion but no arterial invasion and without lymph node involvement. The lesion of segment I was metastatic with large areas of necrosis. The tumor was classified as pT3 N0 M1. The postoperative course was uneventful and the patient was discharged on postoperative day 22. Six cycles of adjuvant chemotherapy (half-dose of GEMOX regimen because of severe thrombopenia) were administered postoperatively (Table 3).

**Table 3:** **Adjuvant chemotherapy regimen for the three patients**

	Patient number 1	Patient number 2	Patient number 3
First-line (adjuvant) chemotherapy	GEMOX (6 cycles)	FOLFOX (6 cycles)	GEMZAR (8 cycles)
Second-line chemotherapy	FOLFOX (8 cycles)	/	/
Third-line chemotherapy	FOLFIRI (6 cycles)	/	/

### Patient number 2

**Figure 2:**
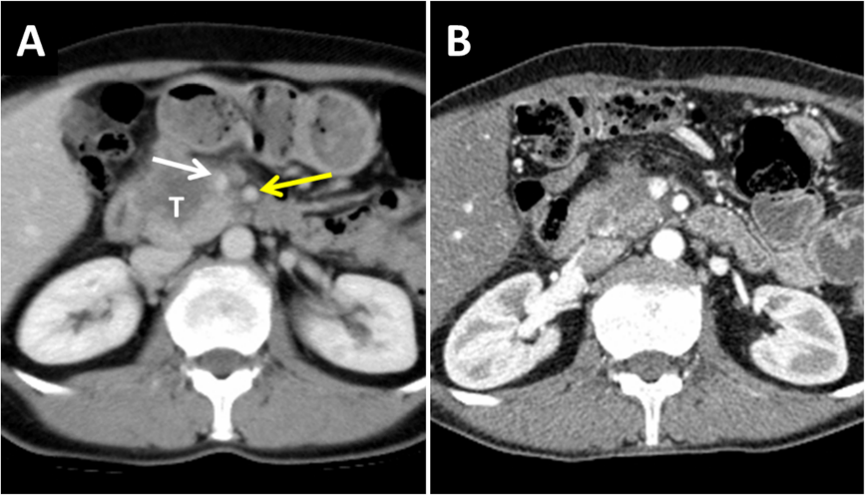
**Computed tomography (CT) scan of patient number 2 before the initial palliative bypass procedure (A) showing a huge tumor of the head of the pancreas (T) with tumoral infiltration of both superior mesenteric vein (SMV) (white arrow) and superior mesenteric artery (SMA) (yellow arrow).** Following six cycles of FOLFIRINOX chemotherapy and bypass procedure, the CT scan showed regression of the infiltration of the SMA with no detectable metastases **(B)**.

Patient number 2 was a 53.5-year-old female. Clinical symptoms were abdominal pain. Preoperative imaging showed a 65-mm lesion of the head of the pancreas with both portal vein and mesenteric arterial involvement. Despite this, she had undergone surgical exploration in a previous center, which confirmed neoplastic portal thrombosis and SMA invasion (Figure 2). Interaortocaval lymph node procurement with intraoperative frozen section showed metastatic lymph nodes with capsular disruption. Resection was abandoned and a double bypass (hepaticojejunostomy and gastroenterostomy) was performed. The patient underwent systemic chemotherapy (six cycles of FOLFIRINOX regimen followed by two cycles of FOLFIRI) after the initial palliative procedure. At the end of chemotherapy, a CT scan showed a dramatic decrease in the size of the tumor (63 mm to 32 mm), loss of the SMA encasement and partial recanalization of the portal vein. Tumor biomarker CA19-9 had returned to normal range (200 to 5 UI/L). The patient had a weight gain of 4 kilograms and was in good general condition. Resection was decided on because of objective response and stable disease after a period of 8 months. PD with portal vein en bloc resection was performed. The conclusion of the final pathologist’s report was PDAC with portal vein invasion without lymph node involvement. Posterior resection margin was <1 mm. The tumor was classified as pT3 N0 M0. The postoperative course was uneventful, and the patient was discharged on postoperative day 12. Six cycles of adjuvant FOLFOX chemotherapy were administered postoperatively (Table 3).

### Patient number 3

**Figure 3:**
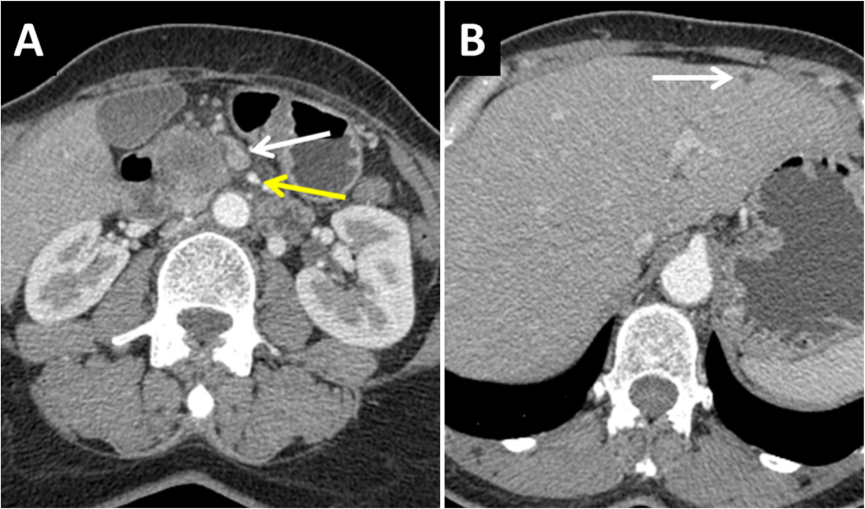
**Computed tomography (CT) scan of patient number 3 before initial palliative bypass procedure showing a tumor of the head of the pancreas (T) with lack of invasion of superior mesenteric vein (SMV) (white arrow) and superior mesenteric artery (SMA) (yellow arrow) (A) but unrecognized subcapsular liver metastases (white arrow) (B).**

Patient number 3 was a 60-year-old female. Clinical symptoms were abdominal pain. Preoperative imaging showed a 38-mm lesion of the head of the pancreas with no vascular invasion and no distant metastases (Figure 3). She had undergone surgical exploration in a previous center. At laparotomy, a suspect lesion in segment III was resected. Intraoperative frozen section showed evidence of liver metastasis from PDAC. Resection was abandoned and gastroenterostomy was performed. The patient underwent postoperative systemic chemotherapy (12 cycles of FOLFIRINOX followed by 7 cycles of gemcitabine). At the end of chemotherapy, a CT scan showed no progression of the disease, and tumor biomarker CA19-9 was within the normal range (34.1 UI/L prior to chemotherapy and 17 UI/L after the two lines of chemotherapy). The patient was in good general condition. Resection was decided on because of stable disease after a period of 14 months. PD with SMV en bloc resection was performed because of suspected adhesions to the SMV. The conclusion of the final pathologist’s report was PDAC without SMV involvement and R0 resection margins. There was no lymph node metastasis. Postoperative course was uneventful, and the patient was discharged on postoperative day 12. Eight cycles of adjuvant gemcitabine chemotherapy were administered postoperatively (Table 3).

### Characteristics of metastases

Metastases were single and hepatic in two cases (patients number 1 and number 3) and interaorticaval in the other (patient number 2). For patients number 2 and number 3, metastases had been resected at the time of primary surgery whereas the single metastasis of patient number 1 was incidentally discovered and resected concomitantly with PD. Liver metastases were <1 cm in the two patients concerned.

### Oncologic follow-up

Median follow-up was 26.3 months (range, 24.7 to 32.5 months). Patients number 2 and number 3 did not experience recurrence until the last follow-up (26.3 and 24.7 months respectively). Patient number 1 had a huge increase in CA19-9 11 months after surgery with no patent target seen on a CT scan or PET scan. Nevertheless, he received six cycles of chemotherapy at half-dose because of thrombopenia (Table 3). A control CT scan showed four liver and one lung metastases. The patient received eight cycles of FOLFOX regimen followed by six cycles of FOLFIRI regimen. He died 2 years after surgery (32 months after the beginning of the disease).

## Discussion

PDAC with synchronous distant metastases has a very poor prognosis and in cohort studies median survival rarely exceeds 6 months [14–16]. New regimens of chemotherapy and radiotherapy has improved the prognosis of metastatic and/or locally advanced PDAC [17, 18], but the benefit of surgery in such cases is still a matter of debate. However, there is robust evidence that some patients with locally advanced or metastatic disease can be cured following aggressive therapy. In the study of Adham and coworkers concerning prolonged survival after resection of PDAC, six patients had advanced disease and one had metastatic disease [19]. Bachellier and coworkers showed that initially advanced disease could be down staged by neoadjuvant chemotherapy, and that in such cases, resection offered a significant prolonged survival, as in patients with initially resectable PDAC [20]. In contrast, Gleisner and coworkers in 2007 reported a series of patients with concomitant resection of pancreatic cancer and liver metastasis [6]. In their series, 17 patients had PDAC, most of them with a single liver metastasis. The authors concluded that there was no benefit to resection of liver metastases compared to palliative care, although 3 year-survival was higher in resected patients (6.7% versus 0%). Finally, it is likely that some patients with metastatic and/or locally advanced PDAC have good molecular behavior and can benefit from aggressive resection. However, the ability to select such patients is currently not possible with standard molecular biomarkers. Furthermore, standard imaging still fails to detect millimetric metastasis that could improve preoperative staging and thus avoid unnecessary resections. In this setting, preoperative chemotherapy acts as a ‘test-of-time’ and can help to select patients with less aggressive PDAC that could benefit from radical surgery, even in case of limited metastatic disease.

Consequently, resection of metastatic PDAC remains challenging and raises three important questions. First, do all types of distant metastases (for example, liver, nodal, and peritoneal) have the same prognosis? PDAC with distant metastases is usually estimated as definitively nonresectable and thus palliative chemotherapy is administered. The results of series of metastatic PDAC have shown such a poor prognosis that the impact of the type of metastasis has been rarely investigated [6]. The AJCC-UICC staging system attributes the item ‘M’ for metastatic disease, but does not take into account the location of the metastases. This problem is the same with the item ‘N’, for which it has been shown that prognosis differs according to the type of lymph node metastases (peripancreatic or mesenteric) [21]. Given that distant metastases have a very poor prognosis, the metastatic site has little impact on therapeutic management. Distant metastases in PDAC are most of the time visceral (liver, lung) or nodal (interaorticaval) [8]. However, despite no survival benefit of extended lymphadenectomy in PDAC [22], metastatic interaortocaval lymph nodes seem to have a better prognosis than metastases in other organs such as the liver and peritoneum [23, 24]. Similarly, local recurrence after initial resection seems to have a better prognosis than metastatic recurrence, and can be managed by surgery with survival benefit in selected patients [25]. Thus, in the event of objective response to chemotherapy, the type of distant metastases should be considered, as in our patient number 2. In such cases, PD with systematic en bloc interaorticaval lymphadenectomy can be proposed since interaorticaval lymphadenectomy does not increase postoperative morbidity [26].

Second, is the number of metastases important? In patients number 1 and number 3 in our series, laparotomy disclosed single liver metastases undetected by preoperative imaging. Prognosis of PDAC with single liver metastases is better than with multiple liver metastases, [27] and a meta-analysis has shown that resection of PDAC with single liver metastases was comparable to resection of PDAC with no evidence of liver metastases [28]. As in our study, documented reports of prolonged survival after resection of liver metastases from PDAC involved single and small metastases, incidentally discovered at laparotomy [6, 7, 19]. PD may sometimes be performed although there are concomitant single liver metastases that were undetectable because of limited exploration of the whole liver. This is consistent with the rapid time of liver recurrence in PDAC after PD [6, 27, 29]. Given that liver metastases have better prognosis than metastases in other sites (locoregional or peritoneal) [30], single and small liver metastases may indicate a low metastatic volume, as in metastatic colorectal cancer [31] and do not have to be considered as definitely palliative, especially in the event of objective response to chemotherapy.

Third, can we manage liver metastases surgically without previous neoadjuvant therapy? In comparison to colorectal liver metastases (CRLM), concomitant resection of single liver metastases with PDAC has two drawbacks: adjuvant chemotherapy in PDAC is not as efficient as in CRLM, and PD is a major procedure with a high risk of morbidity - in particular pancreatic fistula - that increases the risk of recurrence and reduces the likelihood of receiving adjuvant chemotherapy [32]. However, as indicated above, liver metastases in PDAC may not necessarily be a contraindication to surgery. Laurent and coworkers demonstrated that non-colorectal metachronous liver metastases could justify resection leading to increased survival [33]. In contrast, Gleisner and coworkers in 2007 found no benefit of concomitant resection of PDAC with single liver metastases compared to a palliative procedure [6]. Likewise, Takada and coworkers showed no improvement of survival despite aggressive surgery of PDAC with multiple liver metastases [29]. However, in these two latter studies liver resection of synchronous metastases was performed without neoadjuvant chemotherapy, and recurrence occurred less than 4 months after surgery, suggesting a non-controlled disease [6]. As in CRLM, management of liver metastases needs a ‘test-of-time’, to ensure the control of the disease [34]. In this setting, the FOLFIRINOX regimen showed very promising results compared to the low rate of objective response of a gemcitabine-based regimen [17, 35–37]. Additionally, the cost-effectiveness of neoadjuvant treatment seems to be superior to that of a surgery-first approach [38]. Thus, simultaneous resection of PDAC with single liver metastasis - and by extension all types of distant metastases - should be considered in the setting of neoadjuvant chemotherapy, first to assess the response to chemotherapy and second to assess the aggressiveness of the disease. In the event of objective response, surgical resection can be considered in selected patients with low-volume metastatic liver disease and very good general health status.

## Conclusions

Our short series of three observations shows that R0 resection of PDAC with single distant metastases can offer prolonged survival after selection by neoadjuvant chemotherapy. Only patient number 1 experienced recurrence, but this patient had a T4 tumor with a high risk of local recurrence, and subsequent development of liver metastases that occurred far from the initial site of recurrence. The high rate of recurrence of PDAC raises the question of selecting good responders before resection of PDAC on the basis of systematic neoadjuvant chemotherapy in order to improve long-term survival.

## Abbreviations

CRLM: colorectal liver metastases
CT: computed tomography
GEMOX: gemcitabine and oxaliplatin
IPMN: intraductal papillary mucinous neoplasm
PD: pancreaticoduodenectomy
PDAC: pancreatic ductal adenocarcinoma
PV: portal vein
SMA: superior mesenteric artery
SMV: superior mesenteric vein.
